# Nuclear morphometry and ploidy of normal and neoplastic haemocytes in mussels

**DOI:** 10.1371/journal.pone.0173219

**Published:** 2017-03-10

**Authors:** Francesca Carella, Gionata De Vico, Gabriel Landini

**Affiliations:** 1 Department of Biology, University of Naples Federico II, Naples, Italy; 2 School of Dentistry, College of Medical and Dental Sciences, University of Birmingham, Birmingham, B5 7EG, United Kingdom; Australian Museum, AUSTRALIA

## Abstract

Haemic neoplasia (HN) in bivalves has been reported in association with mass mortality events in various species of molluscs. The aim of this work was to quantify the nuclear morphometry and DNA content of neoplastic cells of mussels *Mytilus galloprovincialis* affected by HN using nuclear densitometry in Feulgen-stained preparations. The results were also compared with a population of normal mussel haemocytes. We captured 256 images of 3 different neoplasia stages and 120 images of normal haemocytes; thus, a total of 120,166 nuclei were analysed. We extracted 21 morphological parameters from normal and neoplastic nuclei. Eighteen of these parameters were different (P<0.05). Among those (expressed in pixel units—*inter-pixel distance of 0*.*45 micrometres*—as: normal vs. neoplastic) nuclear area (117.1±94.1 vs. 423.1±226.9), perimeter (44.9±14.0 vs. 79.0±21.3) and (IOD) integrated optical density (13.47±34.5 vs. 177.1±150.8) were relevant features to discriminate between normal and neoplastic cells. Those differences allowed identifying two distinctive populations of neoplastic nuclei, occasionally in the same individuals at a given phase of the disease. Moreover, neoplastic haemocytes in less extended lesions showed a ploidy value of 6.2 n along with the presence of a second population of circulating cells with a DNA content of 10.7n. In samples with moderate disease only one peak at 7n was observed. Finally, in more severe conditions, a further ploidy peak of 7.8n was recorded, accompanied by a shallow but broad peak of 31n. This latter extreme value is thought to be due to the presence of giant multinucleated cells where individual nuclei overlap in space and cannot be discerned individually. Computer-based imaging allowed the direct visualization of the cell populations and simultaneous collection of ploidy data as well as morphological features of nuclei.

## Introduction

Nuclear morphology is an important feature associated with cellular function and disease. Enlargement of nuclei, anisokaryosis, nuclear pleomorphism, changes in chromatin patterns and nucleolar abnormalities are well-recognized features of neoplastic cells in human [1–3] as well as animal tumours [4–7]. The quantitative assessment of variability of nuclear features in pathological and physiological conditions has important diagnostic applications that can be best achieved using morphometric analysis [7–8].

Haemic neoplasia (HN) is a proliferative disorder of bivalve haemocytes first described by Farley [9] in oysters (*Crassostrea virginica* and *Crassostrea gigas*). HN was later observed in twenty additional molluscan species from various ecosystems and geographic areas, including commercially-important species, and reported in populations suffering mass mortality [10–12]. The disease is characterized by the proliferation of atypical cells with a possible haemocyte origin, which morphologically exhibit high nuclear to cytoplasmic ratio, diffuse chromatin patterns and pleomorphic nuclei [9]. In the advanced disease, those cells typically infiltrate tissues and organs [13–16]. Moreover, previous studies on bivalves revealed abnormal DNA content in these neoplastic haemocytes [17–18]. Several hypotheses have been postulated to explain the origin of the disease, including chemical contamination [19–21], retroviral infection [22–24], natural environmental extreme conditions [25] or animal clonal transmission [26]. Bivalve HN was recently proposed as an emerging animal model for human cancer [27], however, there are many aspects of the disease that remain unclear, including tumour histogenesis and cell evolution over neoplastic progression, which appear to be different across the affected species [13]. In the Mediterranean mussel *Mytilus galloprovincialis*, very few cases of HN have been reported to date and therefore the descriptions of the cytological/functional features and ploidy patterns are limited [11,13, 28, 29–32]. In relation to cell types, there has been reported the existence of the so-called A and B cell subpopulations in individuals affected with HN in different mussel species (e.g. *M*. *edulis* and *M*. *galloprovincialis*) under both light and electron microscopy in contrast to flow cytometry [13, 33–34]. These cells, characteristic of haemic neoplasia in mussel species, are easily discernible by their peculiar morphology [13, 33–34], although their role in disease pathogenesis have not been clarified. It has been speculated that A and B cell types might represent two distinct cancer cell lineages, while others suggested they could represent consecutive developmental stages of a single cell lineage during disease progression [13, 34–69]. Broad genomic abnormalities of cancer cells can be reliably studied by both FCM (flow cytometry) and image cytometry (ICM). The latter is often used to perform retrospective studies of DNA quantitation in archived material [37]. The standardized ICM is recognized as a non-subjective, cost-effective and accurate technique to study DNA alterations and ploidy changes [37]. In this context, despite that nuclear morphometry and DNA densitometry have been extensively studied in human neoplasia, there have been few applications in the field of invertebrate pathology. In order to add new insights in the biology of this interesting neoplasia, we focused on HN in *M*. *galloprovincialis* and used a morphometric approach involving light microscopy to 1) describe the nuclear morphology and quantify DNA contents of the neoplastic cells of mussels affected by HN in Feulgen-stained histological preparations; 2) compare the obtained data with normal haemocytes; and 3) assess the data in the context of the disease progression according to staging.

## Materials and methods

### Sampling, histology and feulgen reaction

A total of 35 Mussels (*M*. *galloprovincialis*) specimens were selected and retrieved from histological archival material of the Department of Biological Sciences, University of Naples Federico II belonging to a total sampling of 600 individuals during 2010. Fifteen mussels (2.5% out of 600) were affected by HN and used for the study; an additional 20 unaffected individuals showing normal circulating haemocytes were used as controls. The affected individuals were from 3 mussel farms from the Gulf of Naples (southern Italy, Campania region) obtained in March, May and June 2010: Nisida (N 40°.47985; E14°.9811 7) (6 individuals), Capo Miseno (N 14°.09362; N 40°.78644) (5 individuals) and Castellammare (NO 14°. 41745; 40°.41327) (4 individuals). The healthy individuals (n = 20) were obtained from the Gulf of Salerno (S 14°.51582; S 40°.32779) (southern Italy, Campania region). (As invertebrate animals, no specific permissions are required for mussel sampling/study activities. Moreover we confirm that the field studies did not involve endangered or protected species).

Archived material was processed by routine histological techniques. Briefly, animal shells were opened by severance of the adductor muscle followed by removal of the soft tissues. Three to 4mm thick slices of tissue were sampled along a standard plane; thus, parts of all major organs and tissues (gill, mantle, kidney, digestive gland, gonad and byssus gland/foot) were included in a single histological section. Excised samples were placed into histological cassettes, immediately transferred to buffered formalin 4% for at least 48h, embedded in paraffin blocks and sectioned at 5μm thickness with a rotary microtome. Two consecutive **s**ections for each specimen were stained, one with routine haematoxylin and eosin (H&E) and the other by the Feulgen reaction, and observed under light microscopy.

H&E preparations under light microscopy were used to diagnose the disease: neoplastic cells were typically large, anaplastic cells found in the connective tissue, blood vessels and sinuses of the visceral mass, muscle, and mantle tissue. They featured hyperchromatic and often pleomorphic nuclei containing one or more prominent nucleoli, and they were accompanied by frequent mitotic figures. Two types of neoplastic cells could be distinguished, namely A and B cells following descriptions in [13, 33–34]. In particular, A-type cells are generally ovoid in shape, exhibit marked pleomorphism (polymetrism and polymorphism), with vesicular nuclei and evident nucleoli, while B-type cells are rounded, larger, with nuclei featuring a dense chromatin pattern.

Feulgen reaction for DNA was performed on all the samples (diseased and controls) and analysed for nuclear densitometry (DNA) and nuclear morphometry. The Feulgen reaction protocol was that reported by previous studies on standardization of diagnostic DNA image cytometry [36–37]. Briefly, slices were hydrated in an alcohol series, hydrolysed in 5N hydrochloric acid at room temperature (RT) for 60 min and then stained with Schiff reagent (Bioptica, Italy) for one hour. The sections were then rinsed in three changes of sulphite water (0.5% sodium metabisulphite) of 5 min each followed by two changes of distilled water (5 min each). The sections were then dehydrated in alcohol, cleared in xylene and mounted with Eukitt (Bioptica, Italy).

By means of light microscopy, the mussels *M*. *galloprovincialis* were ranked using a scale according to Lowe and Moore and Galimany and Sunila [34,38] for disease severity as follows: **healthy/normal** was when no neoplastic cells were present; **light** was when few neoplastic cells were observed in the blood vessels and surrounding gonad and digestive tract; **moderate** was when a few neoplastic cells of various types infiltrate the connective tissue of all organs; and **heavy** was when large number of neoplastic cells infiltrated the connective tissue of all organs with loss of tissue architecture.

Feulgen stained images were digitized using an Olympus BX-50 microscope with a 40X objective (n.a. 0.75,) and a digital camera (QImaging Micropublisher 3.3), providing an inter- pixel distance of 0.45 micrometres. Eight individual shots were averaged to reduce random noise in the image sensor, and the background was corrected by the traditional transmittance ratio method [39]. Subsequent imaging procedures were performed using ImageJ version 1.48r16 [40].

### Image analysis densitometry and nuclear morphometry

Feulgen densitometry relies on the principle that the amount of bound stain is proportional to the amount of DNA present (i.e., stoichiometric). In Feulgen staining, DNA quantitation is based on assigning an optical density (OD) (grey level) to each subunit (pixel) of the image and determining the summed OD of pixels for each nucleus in the image [37].

A total of 256 non-overlapping HN images were captured, including images from heavily (n = 135), moderately (n = 67) and lightly diseased (n = 54) samples. In addition, 120 images of normal haemocytes were also obtained. In total, 120,224 nuclei were analysed (normal haemocytes n = 28,141 and neoplastic nuclei n = 92,083). Moreover, a total of 1000 nuclei of A and B cells from selected areas were separately analysed within the neoplastic population (see Tables **1 and 2**).

**Table 1:** List of morphological parameters (DNA content and shape descriptors) used in the study. All units are in pixels (or pixels squared for cell area parameters). Pixels to micrometer factor is 0.624. “None” are dimensionless values.

Parameter	Units	Description
**IOD**	pixels	The sum of the greyscale values in the particle
**GrAverage**	pixels	Average greyscale values in the particle
**Perimeter**	pixels	Perimeter calculated from the centres of the boundary pixels
**Area**	pixels^2^	The area inside the polygon defined by the perimeter
**MinR**	pixels	Radius of the inscribed circle centred at the centre of mass
**MaxR**	pixels	Radius of the enclosing circle centred at the centre of mass
**Feret**	pixels	Largest axis length
**Breadth**	pixels	The largest axis perpendicular to the Feret diameter
**CHull**	pixels	Convex Hull or convex polygon calculated from pixel centres
**MBCRadius**	pixels	Radius of the Minimal Bounding Circle
**AspRatio**	none	Aspect Ratio = Feret/Breadth
**Circularity**	none	Circularity = 4*π*Area/Perimeter^2^, also called form factor
**Roundness**	none	Roundness = 4*Area/(π*Feret^2^)
**Compactness**	none	Compactness: sqrt((4/π)*Area)/Feret
**Solidity**	none	Solidity = Area/Convex_Area
**Concavity**	pixels^2^	Concavity = Convex_Area*–AreaCArea = Area of the Convex Hull polygon
**Convexity**	none	Convexity = Convex_Hull/Perimeter
**Shape**	none	Shape = Perimeter^2^/Area
**ModRatio**	none	Modification Ratio = (2*MinR)/Feret
**Sphericity**	none	Sphericity = MinR/MaxR
**Rectangularity**	none	Rectangularity = Area/ArBBox* ArBBox = Feret*Breadth, area of the box along Feret diameter

**Table 2:** Descriptive statistic (Mean ± SD) of normal and neoplastic cell nuclei with detail on Perimeter, Area and IOD values. Values of 1000 counted neoplastic A and B cells are also reported. *Asterisk indicates mean values of morphological features significantly different (P<0.05) compared with normal haemocytes.

	Normal Haemocytes	Neoplastic haemocytes
**Perimeter**	44.90 ±14.0	79.03±21.3 *
		**A cell:** 58±0.72*
		**B cell:** 70.03±0.08*
**Area**	117.1 ±94.1	423.13±226.9*
		**A cell:** 250±87.2*
		**B cell:** 389.9±125.6*
**IOD**	13.47 ±34.5	177.13±150.8*
		**A cell:** 133±24.2*
		**B cell:** 162.13±12.2*
**GrAverage**	0.1704 ±0.05	0.369±0.211*
		**A cell:** 0.15±0.02*
		**B cell:** 0.41±0.12*
**MinR**	6.283±1.69	8.540±2.44*
		**A cell:** 5.09±1.2*
		**B cell:**9.02 ± 0.01*
**MaxR**	10.65±2.86	14.47±4.08*
		**A cell:** 9.12±1.12*
		**B cell:** 13.17±13.12*
**Feret**	20.49±5.37	27.879±7.69*
		**A cell:** 12.23±11.76*
		**B cell:**26.31±12.56*
**Breadth**	15.20±3.71	21.160 ±5.55*
		**A cell:** 15.32±3.24*
		**B cell:**22.014±9.1*
**CHull**	42.92±12.9	75.99±19.6*1
		**A cell:** 54.12±46*
		**B cell:**73.467±25. *
**MBCRadius**	10.28±2.68	13.977±3.85*
		**A cell:** 8.59±1.24*
		**B cell:**13.64±5.76*
**AspRatio**	1.190±0.42	1.336±0.267*
		**A cell:** 1.25±0.12
		**B cell:**1.242±0.05
**Circularity**	0.5980±0.09	0.807±0.074*
		**A cell:**0.74±0.2
		**B cell:**0.580±0.71
**Roundness**	0.3709±0.140	0.667±0.115*
		**A cell:** 0.623±0.2
		**B cell:**0.497±0.12
**Compactness**	0.8139±0.09	0.8136±0.07
		**A cell:** 0.781±0.02
		**B cell:**0.586±0.55
**Solidity**	0.7527±0.032	0.961±0.03*
		**A cell:** 0.925±0.02*
		**B cell:**0.625±0.01*
**Concavity**	11.080±10.82	18.13±21.2*
		**A cell:** 14.18±8.1*
		**B cell:**6.71±12.1*
**Convexity**	0.860±0.017	0.964±0.015*
		**A cell:** 0.93±0.02
		**B cell:**0.671±0.01
**Shape**	12.065±2.836	15.71±1.701*
		**A cell:** 16.90±1.1
		**B cell:**15.11±1.2
**ModRatio**	0.628±0.1526	0.625±0.13
		**A cell:**0.542 ±0.14
		**B cell:**0.473±0.02
**Sphericity**	0.606±0.1527	0.604±0.13
		**A cell:** 0.53±0.12*
		**B cell:**0.458±0.01*
**Rectangularity**	0.493±0.0482	0.678±0.04*
		**A cell:** 0.527±0.02
		**B cell:**0.471±0.01

The green channel of the 24-bit colour images of Feulgen stained nuclei were combined into a single image stack and converted to a 32-bit greyscale. The green channel includes the absorption peak frequency for the Feulgen–DNA dye complex and provides a convenient method to estimate the DNA contents via the integrated optical density (IOD) of the nuclei. A region of the slide without tissues provided the measure of incident light. The integrated optical density of the image was computed according to the formula [37]:

$$IOD=\sum_{i=1}^{n}-\log_{10}\left(\frac{IFi}{IBi}\right)$$

where *n =* total number of pixels in the nucleus, *IF*_*i*_ = intensity of the *i*^th^ foreground (nuclear) pixel, and *IB*_*i*_ = intensity of *i*^th^ background (clear area) pixel.

The nuclear mask was computed by means of Otsu's thresholding [41] of the green channel image. Incomplete nuclei partially overlapping the image edges were deleted, and a binary watershed separation routine was applied to separate touching nuclei. Finally, non-haemocyte nuclei in tissues were removed by manual image editing before further processing.

In Feulgen DNA densitometry, reference cells from haploid nuclei are required to estimate the DNA quantity. Such a value is generally calculated by the ratio of the relative DNA content in the cells divided by the DNA measurement, based on a previous reference [37]. This value, called C-value, is the amount of DNA contained within a haploid nucleus expressed in picograms. In order to estimate the DNA content of normal and neoplastic nuclei, the following formula was used:

Cs=(IODsx1Cp)/IODp,

$$Cs=\frac{\left(IODs\times 1Cp\right)}{IODp}$$

where Cs = C nuclear DNA content of the sample, Cp = C nuclear DNA content of the primary standard (in pg), IODs = mean of nuclear IOD value of the sample. and IODp = mean of nuclear IOD value of the primary standard. For mussel *M*. *galloprovincialis*, the primary standard value of normal haemocytes is 1C = 0.96 pg.

**Fig 1:**
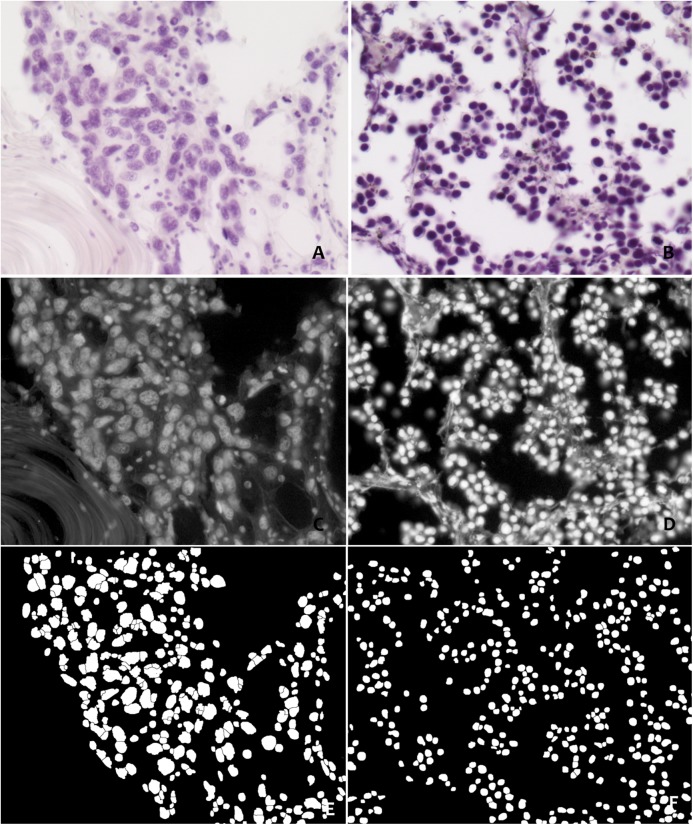
**The sequence of steps to process images of neoplastic nuclei at light disease severity (A, C, E) and heavy (B,D,F) from Feulgen-stained sections (A-B); converted to the green channel (C-D) and after binarisation (E-F)**.

Binary regions that did not correspond to single nuclei were removed by size filtering (between 25 and 300 pixels). In order to determine whether the obtained masks fit the nuclei profiles, we computed the difference between the Feulgen stained images and the binary masks using the *ImageCalculator* of *ImageJ*. The *DilateNoMerge_8* plugin [42] was also used to adjust the nuclear dimension when necessary by morphological dilation without merging with the nearby nuclei. The IOD and associated morphometrical parameters of the nuclei were obtained using the *Particles8* plugin redirected to the 32-bit optical density images [42]. **Fig 1** presents the most relevant steps in the sequence of procedures for image processing (see also **S1 Video File**).

**Fig 2:**
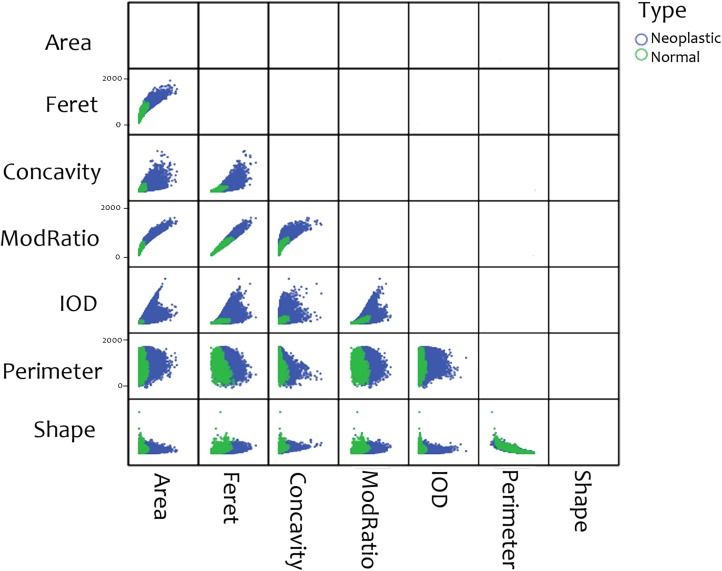
Scatter plots of 7 of the total morphological parameters extracted from normal and neoplastic nuclei.

A total of 21 morphological parameters were measured in the normal and neoplastic nuclei (**Fig 2**, **Table 1**).

### Statistical analysis

The statistical analyses of the data were performed using SPSS 21 (SPSS Inc., Chicago, USA). Descriptive statistics (mean, standard deviation [SD], maximum and minimum) for all the morphological parameters were calculated. The differences between the data groups means were analysed by t test (P<0.05 was considered statistically significant) when only two groups were compared. A regression analysis was computed using a general linear model (GLM) to compare the means of the morphological parameters across the different groups. Stepwise linear discriminant analysis was performed according to normal and neoplastic types and to disease stages. Differences in the distribution shapes of the various parameters were analysed with the Kolmogorov-Smirnov test.

## Results

The mean values of the extracted 21 morphological parameters for normal and neoplastic cells are provided in **Table 2** and **Fig 2.**

**Fig 3:**
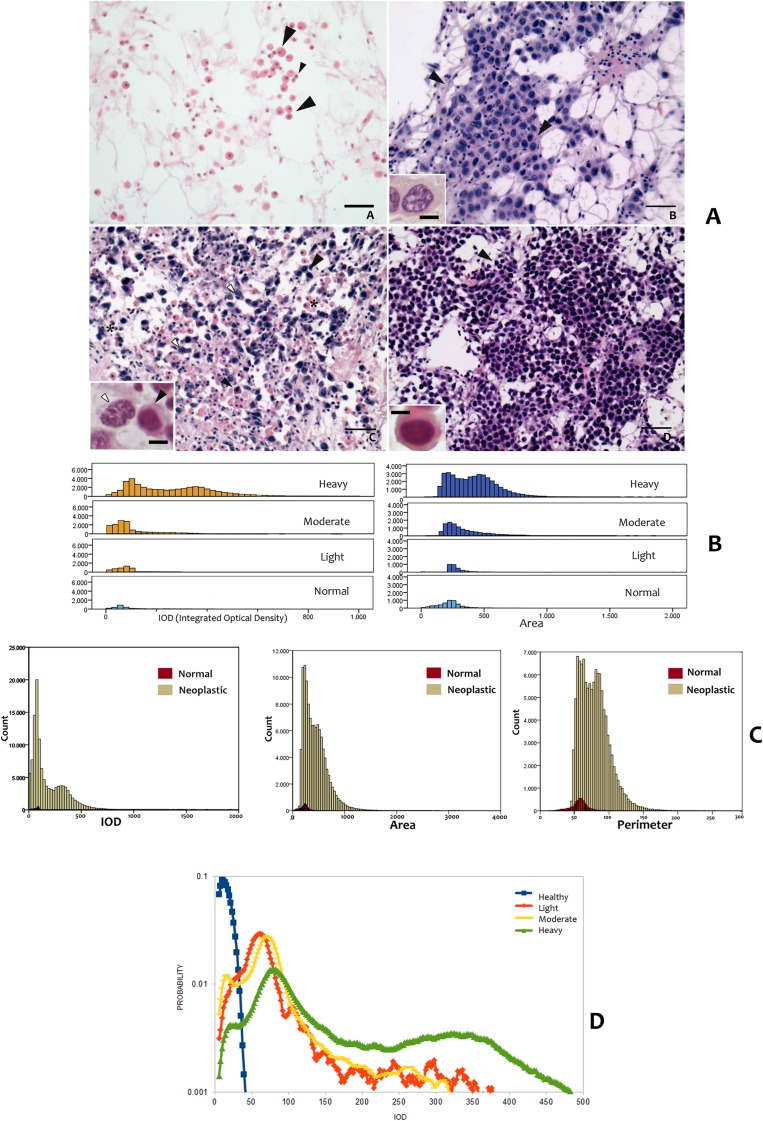
Histopathology coupled with morphometry and ploidy values. **A.** Normal haemocytes and three typical cases of neoplastic condition according to disease severity: A/B cell identification in histological sections and data distribution; **a**: normal haemocytes—granulocyte (big arrowheads) and ialinocytes (small arrowheads); **b:** light disease condition with A cells visible (arrowheads and insert) exhibiting marked pleomorphism (polymetrism and polymorphism) and vesicular nuclei; **c**: moderate disease condition showing presence of A and B cells (insert) mixed with normal haemocytes (*); **d:** heavy disease state primarily exhibits the presence of B cells (insert and arrowheads) rounded in shape, bigger, with nuclei showing a dense chromatin pattern. H&E. Insert Scale bar: 25 μm; **B.** Histograms showing IOD and Area in at different level of disease severity. Notice the second population that arises in the heaviest disease conditions; **(C)** Histograms showing the differences in IOD, Area and Perimeter between normal and neoplastic nuclei; (**D)** Detailed small fluctuations in the IOD distribution over disease progression and compared with normal cells. The data were smoothed with a running average filter of size 7 to preserve the large scale features of the plots.

According to the disease severity, six cases were classified as early lesions (light) with neoplastic cells (A cells only) underlying the stomach epithelium (**Fig 3A and 3B**). Four cases were at the intermediate level (moderate diffusion) of neoplasia characterized by small isolated scattered foci of both A and B cells distributed in different percentages in gills, visceral mass, mantle and kidney (**Fig 3A and 3C**). At this stage, necrosis of digestive tubules was recorded in two cases. The more severe disease condition (5 cases) consisted of massive proliferation of rounded cells (B-cells only) with a dense chromatin pattern, replacing all of the vascular spaces with an extensive loss of tissue architecture (**Fig 3A and 3D)**. The degree of infiltration by the neoplastic cells varied among animals but was most evident in the connective tissue, underlying the stomach and intestine in the digestive gland region. Statistical analysis of the morphometric data by using a multivariate general linear model revealed that the mean values of normal and neoplastic nuclei were significantly different (P<0.05) for most of the morphological parameters, with the exception of the compactness, modification ratio and sphericity (**Table 2**). Interestingly, according to cases and disease severity, data revealed a bimodal distribution possibly indicating a transition from less to more severe lesions, suggested by the IOD and area values (**Fig 3A and 3B**) and (**Table 2**).

Comparisons between the area, perimeter and IOD in normal haemocytes and neoplastic nuclei are presented in **Fig 3C**. Pairwise comparison using the Kolmogorov-Smirnov test showed significant differences in the distribution of the IOD, area and perimeter in normal and neoplastic nuclei (P<0.01).

Within the neoplastic group, the IOD distribution also suggested two different populations of nuclei (appearing bimodal). Two populations were also suggested by the presence of two peaks in the distribution of the nuclear area and perimeter length (**Fig 3C**). The ploidy status of neoplastic nuclei was characterized by different values among the different phases (**Fig 3D**). Normal haemocytes nuclei were considered to have a ploidy value of 1; a peak at this position was also present in all the three disease conditions (light, moderate and heavy). In HN-affected mussels with the light lesions, the ploidy value was 6.2n along with a second aneuploid population at 10.7n. In samples exhibiting moderate disease, only one peak at 7n was observed. Finally, in the heavy category, a peak at 7.8n was recorded accompanied by a shallow but broad peak corresponding to 31n (**Fig 3D**). The possible reason for these disproportionate DNA contents is discussed later.

**Fig 4:**
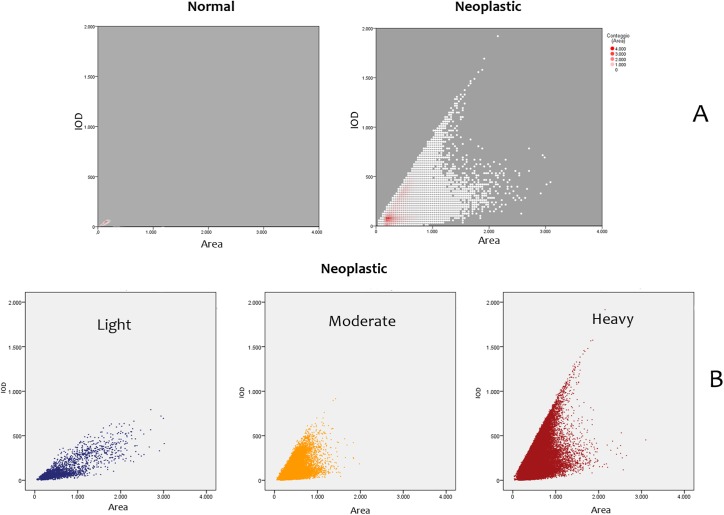
(**A)** Binned scatterplot for area and IOD in normal and neoplastic nuclei: denser area in neoplastic graph is visible at the level of normal haemocytes values for IOD and area. (**B)** Scatterplot of neoplastic samples divided in disease severity.

A scatterplot for the area and IOD showed differences in values in the different disease conditions. Haemocytes of normal appearance were also observed in the diseased samples **(Fig 4A)**. Different values of area and IOD were shown in different disease conditions **(Fig 4B)**.

A hierarchical stepwise linear discriminant analysis using all the cell morphological parameters revealed that 87.6% of cells could be correctly classified as 'normal' and 89.2% as 'neoplastic' (**Table 3**). When classifying neoplastic nuclei according to the current classification of disease severity, the class with the lowest correct classification rate was the light category (42.3% correct) with a large proportion of cases (44.7%) misclassified as moderate (**Table 4**).

**Table 3:** The percentages of correct classification by a stepwise discriminant analysis according to ‘normal’ and ‘neoplastic’ cells.

Original Group	*Predicted group %*		Total
	normal	neoplastic	
**normal**	**87.6**	12.4	100.0
**neoplastic**	10.8	**89.2**	100.0

**Table 4:** The percentages of correct classification by a stepwise discriminant analysis according to disease severity.

Original group	*Predicted group %*				Total
	normal	light	moderate	heavy	
**normal**	**77.8**	0.3	20.3	1.6	100.0
**light**	10.2	**42.3**	44.7	2.8	100.0
**moderate**	15.9	10.1	**59.2**	14.8	100.0
**heavy**	11.2	8.9	27.5	**52.4**	100.0

## Discussion and conclusions

Cell and nuclei shape have long been considered important indicators of the events occurring in the cellular micro-environment [43]. The morphology of neoplastic cell nuclei is currently considered important in the assessment of tumour progression. Nuclear morphometry, i.e., the quantitative assessment of changes in profile, size, shape and optical density is frequently associated with genetic anomalies in cancer cells [44]. Numerous shape descriptors exist in the literature that can in principle be used to characterize various types of cancers and attempt to predict their clinical outcomes [4, 45,46]. Over the years, various efficient quantitative methods of analysis have been developed in the field of microscopy based on mathematical morphology [47], stereology [48] and image processing principles [49]. In addition, image cytometry (ICM) using Feulgen-stained tissue sections has been accepted as an accurate means of quantify DNA contents for clinical applications in human cancer [37, 50–60] and in the determination of animal and plant genome sizes [61,62].

Very few cases of haemic neoplasia of Mediterranean mussel have been described in the literature, suggesting that it might have a low prevalence [13, 29,30,63]. In this work, our data seems to be consistent with the above reports, (2.5% incidence in our samples). From a diagnostic point of view, our results showed that normal haemocyte nuclei were significantly different from neoplastic nuclei, in 18 out of the 21 morphological parameters and this allowed discrimination between the normal and neoplastic cell populations. In particular, nuclear area, perimeter and integrated optical density were the most relevant features for discriminating normal from neoplastic nuclei.

Furthermore, taking into account current theories in the field of carcinogenesis [64], the comparison of morphology and ploidy data as performed here, within the context of tumour progression help us to improve the understanding of the disease pathogenesis. Malignant tumours commonly accumulate genetic defects that promote selection of cell clones with increasing morphological atypia and enhanced ability to invade surrounding tissues and metastasize [64]. Moreover, given that tumours accumulate mutations over time, it is necessary to study neoplastic progression within the temporal context of so-called disease staging, namely the extent of cancer progression at the time of diagnosis. This well-established concept in clinical oncology is reformulated here in terms of light, moderate and heavy disease, depending on the extent of invasion of the host tissues by the neoplastic cells at a given time [65].

Accordingly, in this study, combining morphology and ploidy data, along with the morphological appearance of the cells, support the existence of at least two cell subpopulations that are indistinguishable on a morphological level (e.g. only type-A cells might be detected) but featuring differences in ploidy. These data are in accordance with the notion that neoplastic populations in early stages of cancer are typically affected by genomic instability often resulting in heterogeneous aneuploid cell populations from which, over time, neoplastic clones with higher survival rate and invasive ability emerge [66,67]. In this process, cancer cells typically might undergo alterations in their morphology, occasionally diverging from the original/precursor phenotype as they acquire new genetic and functional changes [66]. A typical example of this process is represented by epithelial cancer cells that are known to acquire a mesenchymal-like phenotype during tumour progression (i.e., epithelial-mesenchymal transition) [68]. In this study, markedly differences in the averages of A and B cell types were observed compared with the overall measures reported of ploidy and morphometry values. This is predictable considering the limited number of counted cells compared with the whole population of neoplastic cells included in the study, also those potentially multinucleated. On the other side, this confirm not only visually, but with morphometric data, the concrete differences between these two cells type. In this context, two hypotheses have been previously suggested regarding A- and B-cell type histo-pathogenesis in *Mytilus sp*. HN. Moore [69] proposed that in *Mytilus edulis*, A and B cell types perhaps represent 2 distinct cell lineages. In contrast, Lowe and Moore [34] and Carella [13] suggested that these cells could represent consecutive developmental stages of a single cell line during disease progression According to the above data, the latter hypothesis seems to be further supported if we consider that in advanced stages of the disease, A-type cells disappear and only B-type cells are observed. According to the data examined in this study and those reported in a recent report [70], co-existence of distinct neoplastic cell subpopulation within the same host in a given time, is typical of intermediate stages of HN in *M*. *gallorprovincialis*. This suggests that the HN pathogenesis in this species could be different from that recently proposed for other bivalves, such as *Cerastoderma edule*, *Mya arenaria* and *Mytilus trossulus*, where a clonal horizontal transmission of neoplastic cells was recently demonstrated [70]. In HN of *C*. *edule*, in particular, two neoplastic cell populations have been described belonging to two distinct neoplastic cell clones (also named Type A and Type B) [70]. The clones are transmitted from affected to healthy animals by horizontal transplantation of neoplastic cells through the sea water. However, the two cell types have never been encountered in the same affected individuals at a given time. In contrast, in *Mytilus galloprovincialis*, A-Type and B-type cells co-exist in a given individual and so they would seem to belong to the same ploidy pool. In *Mytilus trossulus*, a species in which clonal horizontal transmission also occurs, Vassilenko and Baldwin [71] observed ploidy values during tumour progression that differed from those found in the present study and in the studies by Elston [11] and Moore et al [69], suggesting that the diseases in *Mytilus sp*. are perhaps complex and might not exhibit uniformity in their development.

As stated, the morphological changes in neoplastic nuclei also corresponded to an increase in DNA content. In this study we used IOD as a measure of DNA content. Previous comparisons have shown that flow cytometry and image analysis provide similar efficacy of DNA quantification for diagnostic purposes [57]. However, compared with flow cytometry, ICM has a number of advantages: low cost, a small number of nuclei are required and the visual morphological distinction between a cancer and a normal cells (important for cyto-pathologists) is still possible. Because individual cells are analysed, ICM also provides a tool to perform ploidy measurements across cancer cell subpopulations [72]. In our case, ICM led us to measure DNA ploidy in Type-A/Type-B neoplastic cells, suggesting that they belong to a specific ploidy pool, but this could not have been possible using FC, which loses the morphological component of other associated tissue features and requires large sample sizes (>10,000 cells). FC cannot distinguish aneuploidy in a given cell despite the measurement of DNA content per cell because the measurement in FCM is a distribution of DNA content for the entire population of cells [72].

In cancer, the genomic diversity ranges from few to large-scale cytogenetic alterations caused by increased genomic instability [73]. Consistent with this view, DNA content alteration correlates with tumour progression and is observed in solid and haematological malignancies [74]. Similar abnormalities have also been described in HN-affected bivalve molluscs, with differences noted among species [17]. For instance, a study by Farley [9] reported a relatively high proportion of dividing cells with abnormal numbers of chromosomes. Previous studies using flow cytometry (FCM) [34] also reported 2 morphologically distinct types of cells in *M*. *edulis* with different DNA contents along with observations of chromosomal aberrations. These studies reported similar DNA content compared with our cases. With regard to the high values of ploidy observed in our study, [34] an increase in ploidy is noted during the progression of the disease in the neoplastic cell populations. Moreover, this increase in DNA in circulating neoplastic cells occurred in parallel with the morphological changes described in haemocytological preparations. In our study, the peak that was present at every stage could represent the common diploid fraction of cells that were present in every tumour. In addition, the presence of an extremely high value of IOD (31n) at the third stage is unlikely to arise from single cells but perhaps from the effect of nuclear division without cell division, resulting in multinucleated giant cells, as previously described by [13]. Overlapping packed nuclei along the direction of observation might therefore artificially increase the optical density in those multinucleated cells. This possibility might be resolved using transmission electron microscopy to clarify the extent and fine structure of the nuclei in those cells.

Other studies on mussels have linked environmental contamination to DNA damage, including sister-chromatid exchange [75], micronuclei formation [76–77], strand breaks and cross-linking [78–79], G1-arrest and G2-delay [80] and apoptotic processes caused by the environmental pollutant tributyltin [81]. Karyotyping studies have reported the presence of supernumerary chromosomes in cockle (*C*. *edule*) cells from Galicia and Portugal [82]. In a similar manner, karyological analysis of *Mytilus* neoplastic cells demonstrated that the ploidy alteration resulted from extensive chromosomal fragmentation [11]. Moreover, Reno et al [18] observed changes in chromosome shape in *M*. *arenaria*, which may indicate chromosomal breakage.

Stepwise discriminant analysis showed less than a perfect correct classification of cells in the three classes considered. The possibility of an overlap in the populations across the diagnostic classes due to the contemporary presence of various types of neoplastic cells along with normal haemocytes (20.3% at moderate) may explain this finding. Indeed, in the 3-class discrimination, the analysis showed the lowest rate of correct classification for the moderate stage, where different types of cells were present. This underlines the difficulties in finding consistent and unique morphologic classifiers and reveals the complications in the implementation of a standard with regard to neoplasm grading.

In conclusion, our results demonstrate that 1) the quantitative nuclear morphometry provides valuable information to discriminate between normal and neoplastic population of cells in *Mytilus galloprovincialis* HN; 2) the differences observed are statistically significant; 3) the methods also allowed the discrimination of two different populations of cells in the neoplastic samples according to disease severity; 4) the alterations in nuclear structure appear to be closely related to alterations in ploidy, which are also typical of malignant processes.

Quantitative measurements of nuclear features are a pre-requisite to assess physiologic and pathologic responses in model organisms and to enable comparison of data in the accelerating field of experimental animal research [83–84]. Although the observed population of neoplastic cells according to disease progression seemed to be divided into distinct morphological subtypes, the hypothesis of a consecutive developmental stages of a single cell line as hypothesised in [13, 35] is also supported by the ploidy data presented here.

The high values of ploidy found here as well as in a previous study [34] have not been reported in cancer of vertebrates. Perhaps such aberrant genomes are less compatible with cell viability in higher organisms that require complex homeostatic control, although it has been shown that polyploidy can be tolerated in some eukaryotes [85].

Further genomic investigations could better unravel the parental relationship between A-Type and B-Type cells in HN of *M*. *galloprovincialis*; however, present data along with those reported for HN in other species seems to suggest that HN in bivalves should be regarded as a *disease complex* with multiple and different pathogenesis rather than a single entity. Taken together, the results may provide the necessary quantitative baseline for reference in the study of bivalve HN.

## Supporting information

S1 Video File video shows steps necessary to measure ploidy from Feulgen stained preparations. (MP4)
